# Supplementary material for: RNF41 regulates the damage recognition receptor Clec9A and antigen cross-presentation in mouse dendritic cells
Source: eLife. 2020 Dec 2;9:e63452. doi: 10.7554/eLife.63452 (PMC7710356; doi:10.7554/eLife.63452)
Supplement: Supplementary file 1. — Table 2: Glycan modification of Clec9A dimers. Table 3: Proteins that showed an enhanced association with Clec9A in the presence of RNF41. Table 4: Quantification of Clec9A and Ubiquitin ubiquitinated peptides. [file elife-63452-supp1.docx]

**SUPPLEMENTARY TABLES**

**Supplementary Table 1: Identification of human proteins that bind specifically to mouse Clec9A.**

Comparison summary for binding hits of mouse Cire-ecto versus mouse Clec9A-ecto (Z score >3). Signal strength, Z-score and Coefficient of variation were calculated using the Prospector v5 (Life Technologies). A binding hit was defined as any protein where the two replicates on the protoarray slide had an average Z-score of >3 and a coefficient of variation of <0.5. Representative of 2 independent arrays of Cire-ecto and 3 independent arrays with Clec9A-ecto. Only 1 protein (RNF41) was identified that bound to mouse Clec9A but not mouse Cire. Clec9A-ecto did not bind to 29 other RNF family members, including RNF4, RNF5, RNF7, RNF10, RNF11, RNF13, RNF20, RNF32, RNF34, RNF40, RNF111, RNF113A, RNF113B, RNF121, RNF125, RNF126, RNF128, RNF130, RNF138, RNF141, RNF151, RNF170, RNF175, RNF182, RNF183, RNF185, RNF186, RNF190 and RNF208.

| **Database ID** | **Ultimate ORF ID** | **mClec9A Z-Score** | **Cire Z-Score** | **mClec9A CV** | **Cire CV** | **mClec9A Signal** | **Cire Signal** | **Description of Protein** |
| --- | --- | --- | --- | --- | --- | --- | --- | --- |
| **Clec9A-ecto binding hit** | | | | | | | | |
| NM_194358.1 | IOH45538 | 59.32162 | 1.45363 | 0.07016 | 0.08713 | 62253.5 | 3733 | Ring finger protein 41 (RNF41), transcript variant 2, mRNA |
| **Cire-ecto binding hit** | | | | | | | | |
| NM_006819.1 | IOH5061 | 0.08557 | 21.3994 | 0.18511 | 0.1654 | 191 | 37634.5 | Stress-induced-phosphoprotein 1 (Hsp70/Hsp90-organizing protein) (STIP1), mRNA |
| **Clec9A-ecto and Cire binding hits** | | | | | | | | |
| NM_001551.1 | IOH3828 | 62.15254 | 37.22324 | 0.00066 | 0.00072 | 65219.5 | 64530 | Immunoglobulin (CD79A) binding protein 1 (IGBP1), mRNA |
| NM_020166.2 | IOH3833 | 8.50389 | 4.53332 | 0.02542 | 0.03793 | 9011 | 8967.5 | Methylcrotonoyl-Coenzyme A carboxylase 1 (alpha) (MCCC1), mRNA |
| NM_000282.1 | IOH2968 | 26.52978 | 16.21608 | 0.00943 | 0.00101 | 27897 | 28824.5 | Propionyl Coenzyme A carboxylase, alpha polypeptide (PCCA), mRNA |

**Supplementary Table 2: Glycan modification of Clec9A dimers.**

Clec9A dimers were excised from SDS Page gels, subjected to tryptic digest and analysed by LC-MS/MS. N-linked glycan modification of either Clec9A N81 (stalk region) or N159 (CTLD) is shown as mean +/- SD of the mass gain (Da), n represents the number of glycosylated peptides analysed. Glycosylation of the 75 kDa and the 60 kDa forms were analyzed by unpaired t-test.

| **Clec9A dimer form** | **N81 (stalk)** | **N159 (CTLD)** |
| --- | --- | --- |
| **75 kDa** | 2635.43 +/- 439.62 (n=53) | 2261.00 +/- 126.34 (n=6) |
| **60 kDa** | 1728.57 +/- 717.29 (n=28) | 1702.88 +/- 173.45 (n=8) |
| **p-value** | **** p<0.0001 | **** p<0.0001 |

**Supplementary Table 3. Proteins that showed an enhanced association with Clec9A in the presence of RNF41**. 25 proteins that showed over 4-fold increase in association with Clec9A and RNF41 compared with either vector alone or RNF41 ΔRING, and with an FDR cutoff of 0.05, and 2 selected proteins that showed over 2-fold increase in association with Clec9A and RNF41. Data is presented as Log_2_ Fold Change.

| **Gene name** | **Protein names** | **Log_2_ Fold Change Clec9A+RNF41 vs Clec9A** | **Log_2_ Fold Change Clec9A+RNF41 vs Clec9A+RNF41 ΔRING** |
| --- | --- | --- | --- |
| **>4-fold increase in both comparisons** | | | |
| RPL36 | 60S ribosomal protein L36 | 6.30 | 2.38 |
| PLD3 | Phospholipase D3 | 3.51 | 3.90 |
| TMEM59 | Transmembrane protein 59 | 3.27 | 2.87 |
| CPD | Carboxypeptidase D | 3.02 | 2.95 |
| ITM2B | Integral membrane protein 2B | 2.80 | 2.86 |
| SLC3A2 | 4F2 cell-surface antigen heavy chain | 2.61 | 2.62 |
| CALR | Calreticulin | 2.19 | 2.01 |
| NDUFS4 | NADH dehydrogenase [ubiquinone] iron-sulfur protein 4, mitochondrial | 2.03 | 2.07 |
| CYP51A1 | Lanosterol 14-alpha demethylase | 2.03 | 2.76 |
| ERLIN2 | Erlin-2 | 2.01 | 2.13 |
| **>4-fold increase compared to Clec9A alone & >2-fold increase compared to Clec9A +RNF41 ΔRING** | | | |
| GPX1 | Glutathione peroxidase 1 | 2.02 | 1.94 |
| DNAJB1 | DnaJ homolog subfamily B member 1 | 2.28 | 1.80 |
| HLA-A | HLA class I histocompatibility antigen, A-2 alpha chain | 2.07 | 1.81 |
| ADCK3 | Atypical kinase ADCK3, mitochondrial | 2.19 | 1.55 |
| TIMM44 | Mitochondrial import inner membrane translocase subunit TIM44 | 2.21 | 1.32 |
| ***>4-fold increase compared to Clec9A alone & <2-fold increase compared to Clec9A +RNF41 ΔRING*** | | | |
| NUP93 | Nuclear pore complex protein Nup93 | 2.11 | 0.85 |
| RNF41 | E3 ubiquitin-protein ligase NRDP1 | 6.90 | -0.65 |
| CACYBP | Calcyclin-binding protein | 5.24 | -0.01 |
| **>2-fold increase compared to Clec9A alone & >4-fold increase compared to Clec9A +RNF41 ΔRING** | | | |
| PANK4 | Pantothenate kinase 4 | 1.98 | 3.06 |
| CSE1L | Exportin-2 | 1.80 | 3.24 |
| NRBP1 | Nuclear receptor-binding protein | 1.81 | 2.05 |
| DHCR24 | Delta(24)-sterol reductase | 1.65 | 2.79 |
| BAG2 | BAG family molecular chaperone regulator 2 | 1.58 | 2.02 |
| **<2-fold increase compared to Clec9A alone & >4-fold increase compared to Clec9A +RNF41 ΔRING** | | | |
| PSMB1 | Proteasome subunit beta type-1 | 0.79 | 2.33 |
| CYC1 | Cytochrome c1, heme protein, mitochondrial | 0.12 | 2.23 |
| **Selected proteins with an >2-fold increase in both comparisons** | | | |
| CANX | Calnexin | 1.02 | 1.02 |
| SEC61A1 | Protein transport protein Sec61 subunit alpha isoform 1 | 1.81 | 1.27 |

**Supplementary Table 4.** **Quantification of Clec9A and Ubiquitin ubiquitinated peptides.** Quantification results of 6 and 3 ubiquitinated peptides derived from Clec9A and Ubiquitin, respectively, across 3 independent experiments as indicated. The intensities of the ubiquitinated peptides are shown as Log_2_. Empty cells or NaN (Not a Number) indicates that the ubiquitinated peptide was not confidently detected or quantified.

|  | | **Experiment 1** | | | **Experiment 2** | | | **Experiment 3** | | |
| --- | --- | --- | --- | --- | --- | --- | --- | --- | --- | --- |
| Ub Site | Spectra | Clec9A | Clec9A + RNF41 | Clec9A + RNF41 ΔRING | Clec9A | Clec9A + RNF41 | Clec9A + RNF41 ΔRING | Clec9A | Clec9A + RNF41 | Clec9A + RNF41 ΔRING |
| **CLEC9A** | | | | | | | | | | |
| K88 | KYTLEYCQALLQR | 22.29 | 23.19 | 22.22 | 20.20 | 22.10 | 20.42 | 20.86 | 22.21 | 20.68 |
| K167 | SCLKEGASLFQIDSK | 19.79 | 21.83 | 20.97 | 18.15 | 21.81 | 18.57 | 20.34 | 21.56 | 18.26 |
| K178 | EGASLFQIDSKEEMEFISSIGK | NaN | NaN | NaN | NaN | 21.16 | 18.48 | 20.30 | NaN | 19.57 |
| K195 | GGNKYWVGVFQDGISGSWFWEDGSSPLSDLLPAER | NaN | 22.82 | NaN | 22.41 | 22.38 | NaN | 21.97 | 22.12 | NaN |
| K238 | SAGQICGYLK or SAGQICGYLKDSTLISDK | 21.67 | 22.30 | 22.01 | 21.23 | 21.37 | 20.78 | 21.10 | 22.36 | 21.36 |
| K251 | CDSWKYFICEK |  |  |  | NaN | 22.59 | NaN | NaN | NaN | NaN |
| **Ubiquitin (RPS27A/UBA52/UBB/UBC)** | | | | | | | | | | |
| K11 | TLTGKTITLEVEPSDTIENVK | NaN | 21.85 | NaN | NaN | 20.87 | NaN | NaN | 21.25 | NaN |
| K48 | LIFAGKQLEDGR | 22.95 | 24.49 | 22.98 | 21.31 | 22.73 | 20.07 | 20.75 | 23.05 | 20.63 |
| K63 | TLSDYNIQKESTLHLVLR | NaN | 22.36 | NaN | 19.34 | 21.05 | 19.21 | 18.34 | 21.85 | 19.49 |
